# Intravenous versus Partial Oral Antibiotic Therapy in the Treatment of Uncomplicated Bloodstream Infection Due to *Streptococcus* Species

**DOI:** 10.3390/microorganisms11092313

**Published:** 2023-09-14

**Authors:** Lynn E. Broermann, Majdi N. Al-Hasan, Sarah Withers, Kristina L. Benbow, Taylor Ramsey, Meghan McTavish, Hana R. Winders

**Affiliations:** 1 University of South Carolina College of Pharmacy, Columbia, SC 29208, USA; lynn.broermann@va.gov (L.E.B.); kbenbow@email.sc.edu (K.L.B.); taylor.ramsey@va.gov (T.R.); meghanmctavish13@gmail.com (M.M.); 2 Department of Medicine, University of South Carolina School of Medicine, Columbia, SC 29209, USA; 3 Department of Internal Medicine, Division of Infectious Diseases, Prisma Health Midlands, Columbia, SC 29203, USA; 4 Prisma Health Upstate, Greenville, SC 29605, USA; sarah.withers@prismahealth.org; 5 Prisma Health Midlands, Columbia, SC 29203, USA

**Keywords:** antimicrobial stewardship, group A *Streptococcus*, bacteremia, oral step-down, *Streptococcus pneumoniae*

## Abstract

This retrospective cohort study examines effectiveness of partial oral antibiotic regimens in uncomplicated bloodstream infections (BSIs) due to *Streptococcus* species compared to standard intravenous therapy. Adult patients with uncomplicated streptococcal BSIs from April 2016 to June 2020 in seven hospitals in South Carolina, USA, were evaluated. Multivariate Cox proportional hazards regression was used to examine the time to treatment failure within 90 days of a BSI after adjustment for the propensity to receive partial oral therapy. Multivariate linear regression was used to examine the hospital length of stay (HLOS). Among the 222 patients included, 99 received standard intravenous antibiotics and 123 received partial oral therapy. Of the standard intravenous therapy group, 46/99 (46.5%) required outpatient parenteral antibiotic therapy (OPAT). There was no difference in the risk of treatment failure between partial oral and standard intravenous therapy (hazards ratio 0.53, 95% CI 0.18, 1.60; *p* = 0.25). Partial oral therapy was independently associated with a shorter HLOS after adjustments for the propensity to receive partial oral therapy and other potential confounders (−2.23 days, 95% CI −3.53, −0.94; *p* < 0.001). Transitioning patients to oral antibiotics may be a reasonable strategy in the management of uncomplicated streptococcal BSIs. Partial oral therapy does not seem to have a higher risk of treatment failure and may spare patients from prolonged hospitalizations and OPAT complications.

## 1. Introduction

Over 500,000 individuals develop bloodstream infections (BSIs) each year in the United States, resulting in more than 70,000 deaths annually and posing a health risk to many [1]. Beta-hemolytic streptococci, viridans group streptococci, and *Streptococcus pneumoniae* all rank among the ten most common bacteria causing BSIs [2]. Beta-hemolytic streptococci BSI has an overall 30-day case fatality rate of 11% in population-based settings [3]. Utilizing appropriate antimicrobial therapy for the treatment of patients with BSIs is essential for reducing mortality and minimizing complications [4].

In the past, intravenous antibiotics have been considered the preferred treatment for BSIs due to concerns of impaired gastrointestinal absorption and subtherapeutic serum levels with oral antibiotics. Despite differences in bioavailability, partial oral antibiotic therapy may reduce healthcare costs, hospital length of stay (HLOS), and complications associated with intravenous catheters in the outpatient setting [5,6,7,8]. Several studies have demonstrated the lower healthcare costs, infection risk, and shorter HLOS associated with partial oral antibiotic therapy compared to standard intravenous regimens [9,10,11,12].

Oral antibiotics with high bioavailability have demonstrated effectiveness in the treatment of BSIs due to Gram-negative bacilli [13,14]. However, the utilization of partial oral antibiotic therapy for the treatment of streptococcal BSIs is a relatively a new concept, and the susceptibility of *Streptococcus* species to penicillin lends to different oral options than most Gram-negative BSIs. Although oral antibiotics have been used in the treatment of streptococcal BSIs, their specific role in therapy remains unclear [5,15,16,17].

The aim of this retrospective cohort study is to examine the treatment failure and HLOS in patients with uncomplicated *Streptococcus* species BSIs receiving partial oral antibiotic therapy compared to standard intravenous therapy.

## 2. Materials and Methods

### 2.1. Settings

The study was conducted at 7 hospitals within Prisma Health-Upstate in South Carolina, USA. These consist of 1 community-teaching hospital and 6 community hospitals that combine to have over 1000 licensed beds. The study was approved by the Institutional Review Board at Prisma Health.

### 2.2. Definitions

Standard intravenous therapy was defined as the receipt of only intravenous antibiotics for the entire treatment course for the BSI for a minimum duration of 7 days. Partial oral therapy described the transition from intravenous to oral antibiotics within 3 to 9 days from the index BSI for a minimum of 4 days of oral antibiotic therapy [12]. A recurrent BSI was defined as a BSI due to the same genus and species of bacteria that occurred with 90 days of an index BSI. A complicated BSI was defined as persistent positive blood cultures for the same bacteria after 72 h of an index BSI or metastatic sites of infection (e.g., infective endocarditis, septic arthritis, osteomyelitis, etc.). A polymicrobial BSI was defined as the growth of more than one species of bacteria in a blood culture, excluding common skin contaminants (e.g., coagulase-negative staphylococci). Pitt bacteremia score was used to determine acute severity of illness at the time of presentation with a BSI [18]. Early clinical failure criteria (ECFC) were used to determine initial response to antibiotic therapy within 72–96 h of an index BSI [19]. These criteria were derived and validated in patients with a BSI due to Gram-negative and Gram-positive bacteria to predict clinical outcomes such as mortality and HLOS based on initial response to antibiotic therapy [19,20]. ECFC include systolic blood pressure < 100 mmHg or vasopressor use, heart rate > 100 beats per minute, respiratory rate ≥ 22 breaths/minute or mechanical ventilation, altered mental status, and peripheral white blood cell count > 12,000/mm^3^ [19].

### 2.3. Case Ascertainment

This multi-hospital retrospective cohort study evaluated adult patients (≥18 years old) hospitalized with uncomplicated BSIs due to *Streptococcus* species from 1 April 2016 to 30 June 2020. The growth of any *Streptococcus* species in at least one blood culture was required for inclusion. Patients with a growth of viridans group streptococci in one of two sets of blood cultures deemed not clinically significant by the treating physician were excluded. Patients whose antibiotic course did not meet the definition of standard intravenous therapy or partial oral therapy were excluded. Patients with a complicated BSI, recurrent BSI, and polymicrobial BSI were excluded. Patients who expired within 7 days of an index BSI were also excluded to reduce the risk of survival bias.

### 2.4. Statistical Analysis

Descriptive statistics were used to summarize the data: medians and interquartile ranges (IQR) for continuous variables and counts and percentages for categorical variables. To assess the differences in the clinical outcomes between patients treated with standard intravenous therapy and partial oral therapy, the primary endpoint of treatment failure, defined as all-cause mortality or BSI recurrence within 90 days of an index BSI, was evaluated. HLOS was examined as a secondary outcome.

Since treatment allocation was not randomized in this observational cohort study, a propensity score analysis was carried out to adjust for potential variables that likely influenced the clinical decision to use partial oral therapy. Logistic regression analysis was used to identify variables that were independently associated with the receipt of partial oral therapy. First, univariate logistic regression was used to examine clinical and laboratory variables within the first 96 h of a BSI that were associated with the receipt of partial oral therapy. Multiple imputation was used to account for missing variables within 96 h of the index BSI. The multivariate logistic regression model included variables associated with partial oral therapy in a univariate analysis (*p* < 0.05) using backward selection. Odds ratios (OR) with 95% confidence intervals (CI) were reported to describe the strength of association between each variable and the propensity of receiving partial oral therapy.

A Kaplan–Meier survival analysis was used to examine the primary outcome of treatment failure. Patients were followed for 90 days from the index BSI or until death or BSI recurrence. This model allowed for the censoring of patients who were lost to follow-up within 90 days of the BSI on the day of the last healthcare visit. Cox proportional hazards regression was used to examine the risk of treatment failure in patients receiving partial oral and standard intravenous therapy after adjustment for the propensity to receive partial oral therapy. A univariate Cox proportional hazards model was used to examine risk factors for treatment failure. The multivariate Cox proportional hazards model included the variable of interest (partial oral vs. standard intravenous therapy), propensity to receive partial oral therapy, and variables associated with treatment failure in the univariate Cox model (*p* < 0.10) using backward selection. Hazards ratios (HR) and 95% CIs were reported to describe the strength of association between each variable and the risk of treatment failure.

Hospital length of stay (HLOS) was assessed as a secondary endpoint using a multivariate linear regression model. A univariate linear regression model was used to identify the risk factors for prolonged HLOS. The multivariate linear regression model included the type of therapy, propensity to receive partial oral therapy, and variables associated with HLOS (*p* < 0.10) in the univariate analysis. Parameter estimates with a 95% CI described the association between each variable and HLOS.

JMP Pro (version 16.0, SAS Institute Inc., Cary, NC, USA) was used for the statistical analysis. The level of significance for statistical testing was defined as *p* < 0.05 (2-sided) unless otherwise specified.

## 3. Results

### 3.1. Clinical Characteristics

Adults 18 years of age and older who were hospitalized with streptococcal BSIs were included in the screening pool (n = 281). Patients with complicated BSIs (n = 46), those who received antibiotic regimens that did not fulfill the definition of either standard intravenous or partial oral therapy (n = 12), and patients who passed away with 7 days of an index BSI (n = 1) were excluded. Of the 222 remaining patients that were included in the analysis, 99 received standard intravenous therapy and 123 were transitioned to partial oral therapy.

Overall, the most common bloodstream isolates were beta-hemolytic streptococci (87; 39%), followed by *S. pneumoniae* (76; 34%) and viridans group streptococci (59; 27%). The antimicrobial susceptibility results of the *Streptococcus* species bloodstream isolates are shown in Table 1. The baseline characteristics were relatively comparable between the two treatment groups except for the source of BSI, Pitt bacteremia score, and ECFC (Table 2). In the univariate logistic regression model, the respiratory source of the BSI (OR 2.71, 95% CI 1.54, 4.77; *p* < 0.001), Pitt bacteremia score < 4 (OR 4.60, 95% CI 1.96, 10.78; *p* < 0.001), and ECFC < 2 (OR 2.10, 95% CI 1.20, 3.66; *p* = 0.009) were associated with the propensity to use partial oral antibiotic therapy. After adjustments were made in the multivariate logistic regression model, only the respiratory source (OR 3.21, 95% CI 1.75, 5.90; *p* < 0.001) and Pitt bacteremia score < 4 (OR 4.37, 95% CI 1.74, 10.96; *p* < 0.001) were independently associated with partial oral therapy and were included in the propensity score model. An ECFC < 2 (OR 1.79, 95% CI 0.96, 3.32; *p* = 0.07) was not independently associated with receiving partial oral therapy.

### 3.2. Antibiotic Regimens

**Figure 1:**
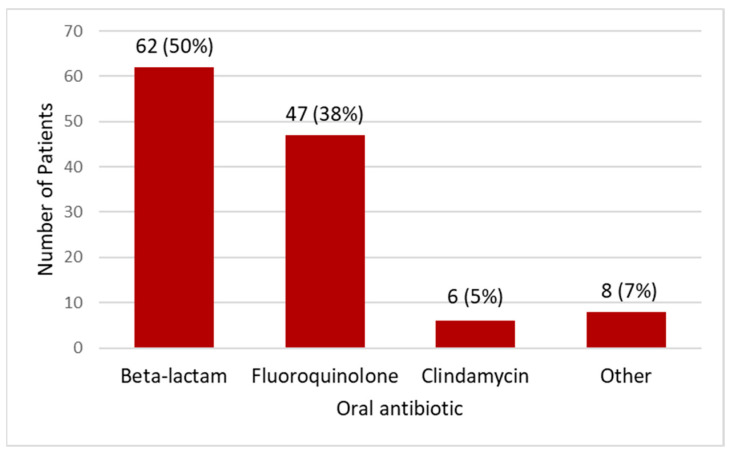
Oral antibiotics used in partial oral therapy group.

The median duration of the total antibiotic therapy was 14 days (interquartile range [IQR] 10–17 days) and 14 days (IQR 12–15 days) in the standard intravenous and partial oral therapy groups, respectively. Patients in the standard intravenous therapy group received a median of 7 days (IQR 5–11 days) of inpatient antibiotics. Almost half of the patients in the standard intravenous group (46; 46.5%) required outpatient parenteral antibiotics therapy (OPAT) after hospital discharge. Patients in the partial oral therapy group received intravenous antibiotics for a median duration of 4 days (IQR 3–6 days) before switching to oral therapy. Beta-lactams (n = 62; 50%) and fluoroquinolones (n = 47; 38%) were the most commonly used oral antibiotic classes in the partial oral therapy group (Figure 1). The less commonly used antibiotics from other classes included clindamycin (n = 6; 5%), trimethoprim/sulfamethoxazole (n = 4; 3%), doxycycline (n = 3; 2%), and linezolid (n = 1; 1%). The oral beta-lactams used included amoxicillin/clavulanate (n = 16), amoxicillin (n = 13), cephalexin (n = 13), cefdinir (n = 9), cefadroxil (n = 6), cefuroxime (n = 3), cefpodoxime (n = 1), penicillin VK (n = 1), and dicloxacillin (n = 1). Levofloxacin 750 mg daily and 500 mg daily were used in 38 and 9 patients in the partial oral group, respectively. *Streptococcus* species bloodstream isolates were susceptible to the oral antibiotics used in the partial oral group. 

### 3.3. Clinical Outcomes

The treatment failure rates were 12.0% in the standard intravenous group and 4.4% in partial oral group, respectively (log-rank *p* = 0.04). All the cases of treatment failure were due to death within 90 days except for one recurrent BSI in the standard intravenous group. The univariate Cox proportional hazards regression model results for the risk factors of treatment failure are presented in Table 3. After adjustments for potential confounding variables and the propensity to receive partial oral therapy in the multivariate Cox model, partial oral therapy was not associated with the risk of treatment failure compared to standard intravenous therapy (HR 0.53, 95% CI 0.18, 1.60, *p* = 0.25). The independent risk factors for treatment failure included older age, cancer, residence at a skilled nursing facility, and higher ECFC (Table 4). 

When stratified by *Streptococcus* species, the partial oral group had a lower treatment failure rate than the standard intravenous group for *S. pneumoniae* (3.8% vs. 20.0%, log rank *p* = 0.02). No difference in treatment failure rate between the partial oral and intravenous-only groups was seen in beta-hemolytic streptococci (4.9% vs. 10.2%, *p* = 0.39) or viridans group streptococci (5.0% vs. 9.4%, *p* = 0.56). The multivariate analyses were not performed on the subgroups due to lack of power.

The median HLOS was 7 days in the standard intravenous group and 4 days in the partial oral therapy group (*p* < 0.001). In the univariate linear regression model, residence at a skilled nursing facility and a higher ECFC were associated with a longer HLOS, whereas partial oral therapy was associated with a shorter HLOS (Table 5). Partial oral therapy was independently associated with a shorter HLOS (−2.23 days, 95% CI −3.53, −0.94; *p* < 0.001) after adjustment for the propensity to receive partial oral therapy among other variables in the multivariate logistic regression model (Table 6).

## 4. Discussion

### 4.1. Growing Evidence for Partial Oral Therapy

In this multi-hospital retrospective cohort study, transitioning patients with uncomplicated streptococcal BSIs from intravenous to oral antibiotics was associated with a shorter HLOS without increasing the risk of treatment failure. These results support a growing shift in favor of partial oral antibiotic therapy for uncomplicated *Streptococcus* species BSIs [15,16,17]. The previous three studies similarly demonstrated shorter HLOSs and a comparable mortality associated with partial oral versus standard intravenous therapy for the treatment of BSIs caused by *Streptococcus* species [15,16,17]. Across all four studies, the most common sources of infection were skin and soft tissue and the respiratory tract [15,16,17]. Given the non-randomized design of these studies, there was a reasonable concern regarding the potential selection of patients with favorable prognoses and a better initial response to antibiotic therapy for transitioning to partial oral regimens [15,16,17]. To our knowledge, the current study was the first to use ECFC to adjust for the initial response to therapy around the time of transitioning from intravenous to oral antibiotics. It was also the first study to use a multivariate model to examine HLOS.

Kang and colleagues demonstrated no difference in the 30-day readmission, mortality, or recurrence rates between treatment groups in a single-center retrospective cohort study of patients with uncomplicated streptococcal BSIs [15]. Moreover, patients who received partial oral therapy had significantly shorter HLOSs compared to those who received standard intravenous therapy. Similar to the current study, most patients in the partial oral group were transitioned to oral fluoroquinolones or beta-lactams after a median of 4 days of intravenous therapy.

Another single-center retrospective cohort of patients with uncomplicated streptococcal BSIs by Ramos-Otero and colleagues demonstrated no difference in 30-day mortality, reinfection at any site, or new-onset sepsis in those receiving partial oral compared to standard intravenous therapy [16]. The study also demonstrated a shorter HLOS and total antibiotic treatment duration in patients transitioned to oral therapy. Almost all patients (94%) in the partial oral group were transitioned to oral beta-lactams, with amoxicillin/clavulanate being the most common agent.

Most recently, a single-center retrospective cohort by Waked and colleagues demonstrated no difference in 90-day mortality or hospital readmission between patients with uncomplicated streptococcal BSIs transitioned to oral antibiotics or continued intravenous therapy [17]. Similarly, the HLOS was shorter in the group that transitioned to oral antibiotics. Among those who transitioned to oral antibiotics, 82% received oral beta-lactams, most commonly cefdinir (25%) and amoxicillin (23%).

### 4.2. Individualizing Treatment Decisions

It should be stated that not all previous studies were in support of partial oral antibiotic therapy for uncomplicated *Streptococcus* species BSIs. A recent investigation by Yetmar and colleagues examining 30-day mortality, relapse, or hospital readmission in BSIs due to beta-hemolytic *Streptococcus* species secondary to a soft tissue source of infection reached a different conclusion [21]. The study demonstrated higher treatment failure rates in patients transitioned to oral antibiotics after nearly 7 days of intravenous therapy as compared to propensity-matched patients who remained on intravenous antibiotics for the reminder of the treatment course. Notably, hospital readmissions from any cause accounted for nearly one-half of treatment failures. Given the relatively high Charlson comorbidity index in this cohort, it is likely that most hospital readmissions were not necessarily related to the index BSIs or respective treatment. Moreover, there was no statistically significant difference in the treatment failure rates at 90 days from BSIs, which was a secondary outcome in the study. It should be noted here that the examination of 90-day clinical outcomes in adult patients with BSIs is recommended by a consensus panel of experts [22]. Aminopenicillins and cephalosporins were by far the most commonly used oral antibiotics in this study. In a subgroup analysis, treatment failure rates were higher in patients who received low-dose as compared to high-dose oral antibiotics [21]. This latter finding was consistent with the results of a previous investigation by Arensman and colleagues [23]. The study found no difference in 90-day mortality, recurrence, or hospital readmission in patients who received fluoroquinolone versus beta-lactam oral step-down therapy for uncomplicated *Streptococcus* species BSIs [23]. However, low-dose oral step-down therapy was a risk factor for clinical failure in the study [23].

Several factors should be taken into consideration when considering transitioning from intravenous to oral antibiotics in patients with *Streptococcus* species BSIs. These include age, comorbidities, source control, early clinical progress, allergies, antimicrobial susceptibility testing results, and potential antibiotic adverse effects. A conservative approach should be taken in patients with advancing age and cancer since these were risk factors for treatment failure in the current study. The optimization of source control (e.g., debridement of soft tissue abscesses, etc.) is preferred prior to the transition from intravenous to oral therapy. An appropriate workup to rule out complications, particularly infective endocarditis, is of the utmost importance in patients with BSIs due to high-risk *Streptococcus* species [24,25]. If oral antibiotics are deemed appropriate in patients with streptococcal endocarditis and other BSI complications, oral antibiotic combinations are preferred, as described in the POET trial and elsewhere [26,27].

### 4.3. Selection of Oral Therapy and Timing of Transition

Regarding the selection of oral antibiotics, when appropriate, levofloxacin 750 mg daily was frequently utilized in patients with uncomplicated BSI due to *S. pneumoniae*. The high susceptibility rates, bioavailability, and probability of achieving target attainment in the serum and lung tissue, which is the likely source of BSIs, support using this agent in patients with *S. pneumoniae* BSIs. The concentration-dependent killing and convenience of once-daily administration, which may improve adherence, make levofloxacin 750 mg daily dose an attractive option to clinicians and patients. On the other hand, aminopenicillins are usually preferred for a BSI due to beta-hemolytic streptococci since these isolates are universally penicillin-susceptible. In our population, 77% of the viridans group streptococci and 99% of the *S. pneumoniae* were also susceptible to aminopenicillins. Based on the results of two previous studies, using high-dose oral aminopenicillins may be preferred [21,23]. The POET trial used amoxicillin 1000 mg four times a day for complicated streptococcal BSIs based on pharmacokinetic and pharmacodynamic parameters [26]. It remains to be determined if this is the optimal amoxicillin dose in uncomplicated beta-hemolytic *Streptococcus* species BSIs. Although oral linezolid was not commonly used in the current or prior studies of streptococcal BSIs [15,16,17,21,23], it remains a viable option given its high bioavailability, prior clinical experience in respiratory and soft tissue sources of infections, and promising results in uncomplicated *Staphylococcus aureus* BSIs [12]. 

The current study provides a time frame based on the clinical pathway for the transition from intravenous to oral antibiotics in *Streptococcus* species BSIs. Patients may be transitioned to oral antibiotics after nearly 4 days of intravenous therapy in those with a favorable initial clinical response to treatment, as determined by ECFC between 72 and 96 h from index BSIs. The ECFC were previously derived and validated to predict mortality and HLOS in patients with Gram-negative and *Enterococcus* species BSIs, respectively [19,20]. In the current study, ECFC ≥ 2 were associated with an increased risk of treatment failure and a longer HLOS. This validates the utility of the ECFC to predict the prognosis of patients with *Streptococcus* species BSIs. It also supports the use of the ECFC as a clinical tool to stratify patients with BSIs who may be considered for transition to oral antibiotics after 96 h of intravenous therapy.

### 4.4. Strengths and Limitations

The use of a propensity score analysis and ECFC to determine the initial response to therapy around the time of transition from intravenous to oral antibiotics represent the major strengths in this investigation. The exclusion of patients who died within 7 days of a BSI reduced the risk of survivor bias. The study shared the common limitations of observational cohorts. Due to the retrospective nature of this study, some missing or unknown confounders may not have been accounted for. The non-randomized design meant that the decision to proceed with oral step-down therapy was made at each providers’ discretion. The adjustment for the propensity to receive partial oral therapy likely reduced the risk of selecting patients with favorable prognoses in the partial oral therapy group. The relatively small sample size did not allow for meaningful subgroup analyses based on BSIs due to a particular *Streptococcus* species. Overall, the low treatment failure rate in the partial oral group precluded the identification of the optimal classes or doses of oral antibiotic regimens. Patients were included from seven hospitals from one healthcare system in South Carolina, USA. This may limit the study’s generalizability to other geographical regions with different patient populations and antimicrobial susceptibility rates. This study included adults with uncomplicated streptococcal BSIs only. The results should not be extrapolated to children or complicated *Streptococcus* species BSIs.

### 4.5. Future Directions

In the short term, a meta-analysis combining the results of the current and previous studies of *Streptococcus* species BSIs may be useful [15,16,17,21,23]. This may overcome the small sample size, which is the biggest limitation of each study. However, it would not necessarily improve the overall quality given the heterogeneity of the studied populations and the various definitions used for the clinical outcomes. A multicenter cohort study using standard definitions based on recommended clinical outcomes may allow for the inclusion of a large number of patients without compromising quality [22]. This may provide adequate power for the examination of partial oral therapy in patients with BSIs due to beta-hemolytic streptococci, *S. pneumoniae*, and viridans group streptococci individually. This large cohort may also provide insights into the most effective oral step-down therapy based on antibiotic class, bioavailability, dose, and the frequency of administration. A randomized controlled trial would be the ideal study design for comparing the safety and efficacy of standard intravenous therapy versus partial oral therapy. The investigators in that trial would have to overcome considerable challenges and barriers, such as time, cost, and other logistical issues related to the enrollment, randomization, and administration of OPAT, when needed.

## 5. Conclusions

There is no difference in the risk of treatment failure in patients with uncomplicated streptococcal BSIs treated with partial oral antibiotic therapy compared to standard intravenous therapy. The early transition from intravenous to oral antibiotics in patients with uncomplicated streptococcal BSIs and favorable initial clinical response to therapy appears to be a safe and effective strategy. Partial oral antibiotic therapy may reduce the healthcare costs associated with prolonged hospitalizations and central venous catheter complications.

## Figures and Tables

**Table 1:** Antimicrobial susceptibility of bloodstream isolates.

Organism	Penicillin	Ceftriaxone	Clindamycin	Erythromycin	Vancomycin	Levofloxacin	TMP-SMZ
Beta-hemolytic streptococci, n = 87, n (%)	87 (100)	87 (100)	50 (57)	45 (52)	87 (100)	NR	NR
*Streptococcus pneumoniae*, n = 76, n (%)	75 (99)	75 (99)	66 (87)	51 (67)	76 (100)	76 (100)	69 (91)
Viridans group streptococci, n = 59, n (%)	43/56 (77)	59 (100)	46 (78)	24/52 (46)	59 (100)	NR	NR

TMP-SMZ: trimethoprim-sulfamethoxazole; NR: not reported.

**Table 2:** Baseline demographics and clinical characteristics of patients with *Streptococcus* species bloodstream infection.

Characteristic	Intravenous Group (n = 99)	Partial Oral Group (n = 123)	*p*-Value
Age, median (IQR)	62 (52–73)	62 (52–71)	0.61
Male, n (%)	47 (47.5)	69 (56.1)	0.20
Race, n (%)			0.67
White	78 (78.8)	91 (74.0)	
African American	17 (17.2)	27 (22.0)	
Other	4 (4.0)	5 (4.1)	
Body mass index, median (IQR)	28 (24–36)	28 (24–37)	0.97
Diabetes mellitus, n (%)	39 (39.4)	46 (37.4)	0.76
Cancer, n (%)	12 (12.1)	11 (8.9)	0.44
Immune compromised host, n (%)	4 (4.0)	6 (4.9)	0.99
End-stage renal disease, n (%)	1 (1.0)	8 (6.5)	0.05
Liver cirrhosis, n (%)	9 (9.1)	10 (8.1)	0.80
Recent hospitalization, n (%)	33 (33.3)	37 (30.3)	0.63
Recent surgical procedure, n (%)	7 (7.1)	10 (8.1)	0.77
Source of BSI, n (%)			<0.001
Skin and soft tissue	31 (31.3)	37 (30.1)	
Respiratory tract	27 (27.3)	62 (50.4)	
Intra-abdominal	2 (2.0)	4 (3.3)	
Other	9 (9.1)	6 (3.9)	
Unknown	30 (30.3)	13 (10.6)	
Pitt bacteremia score, median (IQR)	2 (0–3)	1 (0–2)	0.005
ECFC, median (IQR)	1 (0–3)	1 (0–2)	0.003

IQR: interquartile range; ECFC: early clinical failure criteria.

**Table 3:** Risk factors for treatment failure in univariate Cox model.

Risk Factor	Hazard Ratio	(95% Confidence Intervals)	*p*-Value
Age (per decade)	1.93	(1.33, 2.88)	<0.001
Male sex	0.69	(0.26, 1.85)	0.46
White race	0.92	(0.30, 2.86)	0.89
Body mass index (per point)	1.01	(0.99, 1.03)	0.17
Diabetes mellitus	0.93	(0.34, 2.55)	0.88
Cancer	2.95	(0.95, 9.15)	0.09
End-stage renal disease	1.56	(0.21, 11.79)	0.67
Recent hospitalization	0.70	(0.23, 2.17)	0.54
Residence at skilled nursing facility	8.99	(3.21, 25.94)	<0.001
Recent surgical procedure	0.79	(0.10, 5.97)	0.82
Respiratory source of infection	0.91	(0.33, 2.51)	0.86
Pitt bacteremia score (per point)	1.20	(0.93, 1.48)	0.15
ECFC (per point)	1.31	(0.93, 1.79)	0.10
Partial oral therapy (vs. IV therapy)	0.35	(0.12, 1.01)	0.05

ECFC: early clinical failure criteria; IV: intravenous.

**Table 4:** Independent risk factors for treatment failure in multivariate Cox model.

Risk Factor	Hazard Ratio	(95% Confidence Intervals)	*p*-Value
Age (per decade)	1.60	(1.08, 2.50)	0.02
Cancer	4.76	(1.28, 17.70)	0.03
Residence at skilled nursing facility	7.30	(1.71, 31.27)	0.007
ECFC (per point)	1.56	(1.03, 2.35)	0.04
Propensity of receiving partial oral therapy	0.37	(0.06, 2.29)	0.28
Partial oral therapy (vs. IV therapy)	0.53	(0.18, 1.60)	0.25

ECFC: early clinical failure criteria; IV: intravenous.

**Table 5:** Risk factors for prolonged hospital length of stay in univariate linear regression model.

Risk Factor	Parameter Estimate (Days)	(95% Confidence Intervals)	*p*-Value
Age (per decade)	−0.08	(−0.88, 0.71)	0.84
Male sex	−0.54	(−1.81, 0.73)	0.40
White race	−0.07	(−1.56, 1.42)	0.92
Body mass index (per point)	−0.04	(−0.12, 0.05)	0.39
Diabetes mellitus	−0.19	(−1.50, 1.11)	0.77
Cancer	−0.10	(−2.19, 1.98)	0.92
End-stage renal disease	−1.32	(−4.55, 1.89)	0.42
Recent hospitalization	0.76	(−0.60, 2.13)	0.27
Residence at skilled nursing facility	3.00	(0.31, 5.68)	0.03
Recent surgical procedure	−0.49	(−2.87, 1.90)	0.69
Respiratory source of infection	0.14	(−1.16, 1.44)	0.83
Pitt bacteremia score (per point)	0.58	(−0.11, 1.26)	0.10
ECFC (per point)	2.06	(1.15, 2.97)	<0.001
Partial oral therapy (vs. IV therapy)	−2.83	(−4.05, −1.61)	<0.001

ECFC: early clinical failure criteria; IV: intravenous.

**Table 6:** Predictors of prolonged hospital length of stay in multivariate linear regression model.

Risk Factor	Parameter Estimate (Days)	(95% Confidence Intervals)	*p*-Value
Residence at skilled nursing facility	2.91	(0.37, 5.44)	0.02
ECFC (per point)	1.78	(0.87, 2.69)	<0.001
Propensity of receiving partial oral therapy	0.98	(−6.34, 8.31)	0.79
Partial oral therapy (vs. IV therapy)	−2.23	(−3.53, −0.94)	<0.001

ECFC: early clinical failure criteria; IV: intravenous.

## Data Availability

Data are available upon reasonable request.
